# Association between serum 25(OH)D and death from prostate cancer

**DOI:** 10.1038/sj.bjc.6604865

**Published:** 2009-01-20

**Authors:** S Tretli, E Hernes, J P Berg, U E Hestvik, T E Robsahm

**Affiliations:** 1 The Cancer Registry of Norway, Institute of Population-based Cancer Research, Oslo, Norway; 2 Department of Community Medicine and General Practice, NTNU, Trondheim, Norway; 3 The Norwegian Radium Hospital, Montebello, Norway; 4 Hormone Laboratory, Aker University Hospital, Oslo, Norway; 5 Faculty Division Ullevål University Hospital, University of Oslo, Oslo, Norway

**Keywords:** 25(OH)D, serum, prostate cancer, prognosis, mortality

## Abstract

Based on observations that for certain cancers, mortality varies according to sun exposure, vitamin D has been proposed to influence on disease progression. This study aims to investigate whether serum levels of 25(OH)D are associated with prognosis in patients with prostate cancer. In total, 160 patients with a serum sample in the JANUS serum bank were included. For 123 patients a pre-treatment serum sample was taken, whereas 37 of the patients had received hormone therapy prior to the blood collection. The serum level of 25(OH)D was classified as low (< 50 nmol l^−1^), medium (50–80 nmol l^−1^) or high (>80 nmol l^−1^). A Cox proportional hazard regression model was used to assess the association between serum 25(OH)D and cancer mortality. During follow-up, 61 deaths occurred, of whom 52 died of prostate cancer. The median time of follow-up was 44.0 months (range, 1.2–154.6). Serum 25(OH)D at medium or high levels were significantly related to better prognosis (RR 0.33; 95% CI 0.14–0.77, RR 0.16; 95% CI 0.05–0.43) compared with the low level. Analysis restricted to patients receiving hormone therapy gave a stronger association. The serum level of 25(OH)D may be involved in disease progression and is a potential marker of prognosis in patients with prostate cancer.

The prehormone vitamin D is well known for its important role in calcium regulation and mineralisation of the bone. However, during the last two decades accumulating evidence suggests that vitamin D also influences several other biological processes. As early as 1941, an inverse association between ultraviolet radiation and cancer mortality was suggested (Apperly, 1941). By 1980, the hypothesis that vitamin D deficiency resulting from insufficient sun exposure increased mortality from colon cancer was proposed (Garland and Garland, 1980). The mortality rates for cancer of the breast, prostate, lung, skin and lymphoma have subsequently been shown to vary according to variation in sun exposure (John *et al*, 1999; Freedman *et al*, 2002; Grant, 2002; Robsahm *et al*, 2004; Berwick *et al*, 2005; Porojnicu *et al*, 2005, 2007). Results from recent studies indicate that vitamin D also might influence cancer incidence (*see* Giovannucci, 2005) through mechanisms that influence cancer development and progression and less likely as a part of the cancer initiation. Vitamin D deficiency is suggested to be a risk factor for prostate cancer (Schwartz and Hulka, 1990; Luscombe *et al*, 2001). The effect of vitamin D on cancer processes has repeatedly been demonstrated in experimental studies (*see* Holick, 2006) as it regulates cell cycle processes such as proliferation, apoptosis and angiogenesis in different tissues.

Although several factors influence the level of circulating vitamin D, skin exposure to sunlight is the most important factor. The main dietary contributors include fatty fish, cod liver oil, eggs, and vitamin D fortified dairy products. The biologically most active form of vitamin D, calcitriol (1,25(OH)_2_D), is formed from calcidiol (25(OH)D) in the kidney and is kept at a virtually constant level in serum by parathyroid hormone. However, local production of 1,25(OH)_2_D occurs in different tissues including prostate cells (Schwartz *et al*, 1998), where it regulates key processes as cell differentiation and proliferation (Hansen *et al*, 2001; Omdahl *et al*, 2002). 1,25(OH)_2_D acts by binding to nuclear vitamin D receptors (VDR), and regulates gene transcription. The amount of vitamin D available in the body is closely associated to the concentration of the vitamin D metabolite, 25(OH)D, in the blood. Thus, measurement of circulating 25(OH)D is the best method to estimate vitamin D availability in the body (Freedman *et al*, 2007).

During the winter months at northern latitudes, there is an insufficient amount of UVB in the sunlight to generate vitamin D production in the skin. In Norway, the daily maximum UV-indices vary from zero during the winter months (November–February) to 4.5–6.5 (possible range 0–10) in the mid-summer (Johnsen *et al*, 2002). Similarly, the level of 25(OH)D vary throughout the year, with a mean in the Norwegian population of about 50 nmol l^−1^ during the winter to about 70–80 nmol l^−1^ during the summer (Moan and Porojnicu, 2006). By using season of diagnosis as an indicator for the level of vitamin D, we previously have investigated a possible relationship between vitamin D and cancer-specific survival. Patients who were diagnosed with cancer of the breast, colon, prostate, lung or lymphoma during summer or autumn were found to have better prognosis (15–50%) than patients diagnosed during the winter months (Robsahm *et al*, 2004; Porojnicu *et al*, 2005, 2007). Similar results were observed in a recent study from the UK, which included more than one million cancer patients (Lim *et al*, 2006). A main weakness in these studies is the ecological study design, only using season of diagnosis as a proxy of vitamin D.

This study investigates the relationship between vitamin D and prognosis in patients with prostate cancer, using individual serum levels of 25(OH)D. The results are discussed in terms of the hypothesis that vitamin D suppress cancer processes.

## Materials and methods

The JANUS serum bank was established in 1973, and has serum samples from more than 330 000 healthy donors. Donors who developed cancer and were admitted to the Norwegian Radium Hospital (NRH) for treatment, were asked to donate an additional serum sample to JANUS. The NRH has national responsibilities for specialist medicine, including oncology.

This study is based on 160 patients with histologically verified prostate cancer, diagnosed during the period 1984–2004, who donated serum samples to JANUS. When admitted to the NRH for treatment of their prostate cancer these patients donated a second serum sample to JANUS, which was the inclusion criterion of this study. The serum samples were collected within 30 days of hospitalisation at the NRH, and all patients were alive at least 30 days after the date of serum collection. We had no access to the blood sample taken prior to the cancer disease. A linkage between the Cancer Registry of Norway and JANUS was made through the unique personal identification number (PIN) that identifies each Norwegian citizen. Through this PIN patients were linked to the National Death Registry, which provided information on the date and cause of death up to and including 31 December 2005.

Seventy-five microlitres of serum was drawn from each patient's sample for the 25(OH)D analyses. The level of circulating 25(OH)D was measured using a competitive radioimmunoassay (RIA) (DiaSorin, Stillwater, MN, USA). The RIA is a combined measure of 25-hydroxyvitamin D_3_, and 25-hydroxyvitamin D_2_, which have similar biological activities. The total analytical coefficient of variation was between 13–16% for low, medium and high levels of 25(OH)D. Before any further statistical analyses were performed, the patient's serum level of 25(OH)D was categorised as follows: low (below 50 nmol l^−1^), medium (50–80 nmol l^−1^), and high (above 80 nmol l^−1^). Clinical information about tumour differentiation grade (WHO criteria) (Mostofi, 1980) and functional status at time of hospitalisation at the NRH, as well as treatment information, was retrieved from the medical records (NRH). Functional status was defined as good if performance status, defined by Eastern Cooperative Oncology Group (Oken *et al*, 1982), was ⩽2 and less good if the status was 3 or 4. Patient information from the medical records was collected without the knowledge of the individual 25(OH)D level.

The patients were split into two groups. Patient group I consists of 37 patients, who were diagnosed with prostate cancer at another hospital before the blood donation to JANUS at the NRH. All these patients were on hormonal treatment when entering the NRH. The average time between diagnosis and time of 25(OH)D measurement was 2.4 years.The 123 patients included in patient group II donated the serum sample to JANUS at the time of diagnosis, prior to any treatment. After blood sampling all these patients received treatment: radio therapy (*n*=20), surgery (*n*=29), unspecified treatment (*n*=14) or hormones (*n*=60). Altogether, 97 of the 160 patients received hormone therapy. The hormone therapy given was luteinising hormone-releasing hormone (*n*=54), orchiectomi (*n*=26), anti-androgen (*n*=9) or other hormones (*n*=8). Four patients received both radiation and hormone therapy.

The patients were followed from the date of 25(OH)D measurement until date of death, migration or to the end of follow-up (31 December 2005), whichever occurred first. Cox proportional hazard regression models (SPSS for Windows, Version 15.0, 2007) was used to assess the relationship between serum 25(OH)D level and risk of death from prostate cancer (cause-specific mortality) and death from all causes (mortality).

## Results

### Cause-specific mortality

During the time of follow-up, 61 patients died, of whom 52 died from prostate cancer. The median time of follow-up was 44.0 months (range, 1.2–154.6). Patient characteristics (age, tumour differentiation grade, functional status, serum level of PSA) and 25(OH)D measured at the time of hospitalisation at the NRH are presented for all patients, and for patient group I and II separately in Table 1.

The upper part of Table 2 shows the relationship between 25(OH)D levels and the risk of death from prostate cancer (group I+II). The hazard ratios for patients with medium and high levels of serum 25(OH)D were 0.48 (95% CI: 0.24–0.97) and 0.34 (95% CI: 0.15–0.77) respectively, compared with the patients with low serum levels (Model I). For Model II, when patient group and age were included in the model, patient group status was significant, but did not influence the estimated effect of 25(OH)D. On adding tumour differentiation grade and the patient functional status at the time of blood collection (Model III) a stronger association between 25(OH)D level and the risk of death from prostate cancer was observed.

During the time of follow-up, 33 deaths occurred in patient group I, all due to prostate cancer. The relative risks of dying were 0.31 (95% CI: 0.13–0.73) and 0.26 (95% CI: 0.10–0.68) for patients with medium or high serum levels of 25(OH)D, respectively, compared to patients with low 25(OH)D levels (Table 2, Group I). The results were not substantially altered when age at diagnosis was included (Model II), but the estimated effect was not significant after adjusting for tumour differentiation grade and functional status (Model III).

The last part of Table 2 shows the corresponding results for patient group II. During follow-up, only 19 patients died of prostate cancer. When the analysis was adjusted for age (Model II), tumour differentiation grade and functional status (Model III) a non-significant lower risk of death from prostate cancer was observed for patients with high 25(OH)D values.

All 37 patients in group I and 60 of the patients in group II had received hormone therapy. These 97 patients, of whom 45 died of prostate cancer, were pooled in a separate analysis (Table 3). The mean level of 25(OH)D at blood sampling was 70.7 (s.d.=25.7) in this group compared with 76.0 (s.d.=28.1) nmol l^−1^ in the group not receiving any hormones. When age at diagnosis, functional status and tumour differentiation grade were included in the analysis, the relative risk of dying from prostate cancer was 0.18 (95% CI: 0.07–0.46) for patients with medium levels of 25(OH)D compared with patients with low levels. For patients with high serum 25(OH)D, the corresponding result was 0.09 (95% CI: 0.03–0.27).

### Overall mortality

Sixty-one patients died during follow-up, the majority from prostate cancer. The association between the 25(OH)D level and death from all causes is similar as for cause-specific mortality and is presented in Table 4 (*n*=160).

## Discussion

Ecological studies provide some evidence of a relationship between insufficient 25(OH)D levels and cause-specific mortality in patients with prostate cancer (Robsahm *et al*, 2004; Lim *et al*, 2006), and if substantiated, this may be of importance for the treatment of prostate cancer patients. The present association study shows a rather strong relationship between serum levels of 25(OH)D and cause-specific mortality, especially in the group of patients who received any type of hormonal treatment.

The strength of this association study is that the observations are on an individual level and in a relatively young study population. The median age is 64.5 years, which is about 10 years younger than the median age at diagnosis for prostate cancer in Norway. The young age occurs due to the JANUS blood collection criteria, which allows only patients who previously have donated serum to the JANUS serum bank, to be included. The patients relatively of young age might be an advantage, as they may be less likely to be hospitalised due to health problems related to old age. Furthermore, there is no reason to believe that the inclusion criteria into the cohort or other methodological issues are influenced by the patients’ 25(OH)D level.

A weakness of the study is that some of the patients (group I) have received hormonal treatment prior to the serum sampling. Although it is possible that the treatment itself could affect the patients’ 25(OH)D level, no studies, to our knowledge, have shown that androgen deprivation therapy can influence the 25(OH)D level (Greenspan, 2008).

Prostate-specific antigen (PSA) is established as a diagnostic and prognostic factor within prostate cancer. In this study, information about individual serum levels of PSA was obtained from the patient medical records. All the patients had high PSA-levels (median 17.7 *μ*g l^−1^). The inclusion of PSA in the analyses did not affect the estimated hazard ratios when differentiation grade was included in the analyses (not illustrated). Woo *et al* (2005) observed in a pilot study that a high level of 1,25(OH)_2_D prolongs the doubling time of PSA, which is in line with our results. Unfortunately, in this study we have no repeated measurements of PSA, and hence we were not able to investigate the possible relationship between 25(OH)D levels and the doubling time of PSA.

For each patient the 25(OH)D was measured only once; at the time of hospitalisation. The predictive value of these measurements will depend on the stability of the 25(OH)D by time. In a reliability study of biomarkers Al-Delaimy *et al* (2006) show that the Spearman's rank correlation coefficients were 0.68 and 0.58 for 25(OH)D measurements taken at 5-year intervals for men and women, respectively. The authors conclude that no substantial changes in the mean levels occur over time. Also the time between blood sampling and measurement could create noise in the analyses. Lissner *et al* (1981) have demonstrated a high stability of frozen human blood serum, under several different conditions. Further, Tuohimaa *et al* (2004) claim that storage time does not influence the vitamin D values in their study. Most of their samples were from JANUS serum bank and hence treated in the same manner as the samples in this study.

The disease may, directly or indirectly, have an impact on the 25(OH)D level if patients with advanced disease were less able to attend outdoor activities or have unsatisfactory dietary habits with respect to 25(OH)D. This could be the situation in patient group I, where almost all the patients had metastatic disease at the time of blood sampling, and where 33 patients died of prostate cancer during follow-up. However, in this group the median 25(OH)D level was 62.0 nmol l^−1^, which is a rather high level and not very different from the level in the patients with a non-advanced disease at the time of blood sampling (*n*=114; median level 73.0 nmol l^−1^). Thus, the disease does not seem to have a strong impact on the patient's 25(OH)D level.

In accordance with our hypothesis, the observed association between 25(OH)D levels and the mortality rate can express a beneficial effect of holding 25(OH)D levels above a threshold level (50 nmol l^−1^). No dose–response relationship was seen. The differences between the estimates for medium and high serum 25(OH)D were not significant. Although the major part of the patients included had adequate levels of 25(OH)D, according to recommendations for bone health, we do not know whether there is an optimal level with respect to the prostate health. In this small study including 160 patients in different stages of the disease at blood sampling, the ranges for the 25(OH)D levels were set to ensure reasonable group sizes without knowledge about the patient's serum level.

There is no general consensus on the optimal level of vitamin D for maintaining health (Wolpowitz and Gilchrest, 2006). Some reviews and meta-analyses indicate that there are different thresholds for various health outcomes (Bischoff-Ferrari *et al*, 2006; Vieth, 2006; Gorham *et al*, 2007; Holick, 2007). In this connection results from two incidence studies should be mentioned: Tuohimaa *et al* (2004) demonstrated a J-shaped dose–effect relationship between vitamin D and prostate cancer incidence. A recent study by Ahn *et al* (2008) shows that a high circulating 25(OH)D level may be associated with an increased risk of aggressive disease. Both studies indicate that high serum levels of 25(OH)D can be problematic and thus, the general communication of the result in this study should be done with caution.

The plausibility of an effect of 25(OH)D exists. 25(OH)D can act as a substrate for the 1,*α*-hydroxylation and production of 1,25(OH)_2_D in the prostate or 25(OH)D might bind itself to the VDR in the prostate cells and act by itself (Schwartz, 2005). Several experimental studies have demonstrated the anti-invasive and anti-metastatic effect of vitamin D on prostate cells, through its promotion of differentiation and apoptosis and its inhibiting effect on angiogenesis and proliferation (*see* Dunlap *et al*, 2003). Our results are consistent with the ecological studies showing the lowest death risk among patients diagnosed in seasons with high ultraviolet radiation exposure (Robsahm *et al*, 2004; Lim *et al*, 2006). These results from ecological studies are not in accordance with an influence of the prostate cancer disease on the level of vitamin D. The results in the present study are also consistent with an inverse relationship between plasma 25(OH)D levels and mortality that have been reported for early-stage lung cancer and for colorectal cancer (Zhou *et al*, 2007; Ng *et al*, 2008).

It has been hypothesised that vitamin D can amplify the effect of cancer treatment; a synergistic effect that has been observed in both experimental and clinical studies (Dunlap *et al*, 2003; Deeb *et al*, 2007). Furthermore, results from experimental studies also indicate an interaction between sexual hormones and vitamin D metabolism (Nyomba *et al*, 1987; Sarem and Pedersen, 1988; Bolland *et al*, 2007). The androgen testosterone is synthesised from cholesterol and has a similar structure as vitamin D, and acts through nuclear receptors that belong to the same chemical family as VDR. Thus, a possible explanation for the observed association between 25(OH)D levels and mortality in group I (Table 2) and for the patients on hormone therapy in groups I and II combined (Table 3) might be that 25(OH)D amplifies the therapeutic effects of lowering androgen levels and/or activity and hence improve the cancer prognosis. Among the patients treated without hormones only seven patients died of prostate cancer. This makes it impossible to make an appropriate survival analysis for other treatments than hormone therapy. The different results between group I and II (Table 2, model III), might be due to a difference in the stage of disease. In group II only 19 deaths occurred during follow-up, which indicate the need for longer follow-up or new larger studies.

To conclude, this study shows a strong association between 25(OH)D levels and cause-specific mortality in patients with prostate cancer. The strength of the association indicates that prostate cancer patients can benefit from increasing the level of serum 25(OH)D if it is below 50 nmol l^−1^. However, association studies do not set out to prove causality and several uncertainties exist in the present study. A randomised trial should be carried out, giving the patients vitamin D in addition to the standard cancer treatment.

## Figures and Tables

**Table 1:** Patient clinical characteristics at hospitalisation at the NRH for all patients and patient group I and II separately

**Characteristics**	**No.**	**Median**	**Range**	**Frequency (%)**
*Group I*+*II*	160			
Age at diagnosis	160	64.5	52–82	
Differentiation grade^a^	160			
High	35			17.5
Moderate	97			60.6
Low	28			21.9
Calcidiol nmol l^−1^	160	72.0	19–162	
Low (<50)	29			18.1
Medium (50–80)	82			51.3
High (>80)	49			30.6
Functional status	158			
Good	145			91.8
Less good	13			8.2
				
*Group I*	37			
Age at diagnosis	37	66.7	52–79	
Differentiation grade^a^	37			
High	2			5.4
Moderate	18			48.7
Low	17			45.9
Calcidiol nmol l^−1^	37	62.0	25–135	
Functional status	37			70.3
Good	26			29.7
Less good	11			
				
*Group II*	123			
Age at diagnosis	123	65.7	54–82	
Differentiation grade^a^	123			
High	33			26.8
Moderate	79			64.2
Low	11			9.0
Calcidiol nmol l^−1^	123	72.0	19–162	
Functional status	121			
Good	119			98.3
Less good	2			1.7

a Differentiation grade of tumour tissue; WHO three-grade system.

**Table 2:** The estimated relationship between serum calcidiol and death from prostate cancer for all patients and patient group I and II separately

**Variables**	**Model I RR (95%CI)**	**Model II RR (95%CI)**	**Model III RR (95%CI)**
*Group I*+*II (n*=*160)*			
Calcidiol (nmol l^−1^)			
Low (<50)	1.00 (ref)	1.00 (ref)	1.00 (ref)
Medium (50–80)	0.48 (0.24–0.97)	0.41 (0.20–0.85)	0.33 (0.14–0.77)
High (>80)	0.34 (0.15–0.77)	0.22 (0.09–0.53)	0.16 (0.05–0.43)
Group status		0.05 (0.03–0.10)	0.05 (0.03–0.10)
Age (1 year)		1.00 (0.96–1.03)	1.00 (0.95–1.03)
Differentiation grade^a^			
High			1.00 (ref)
Moderate			1.56 (0.42–5.06)
Low			7.01 (1.91–25.7)
Functional status			
Good			1.00 (ref)
Less good			1.22 (1.00–1.50)
			
*Group I (n*=*37)*			
Calcidiol (nmol l^−1^)			
Low (<50)	1.00 (ref)	1.00 (ref)	1.00 (ref)
Medium (50–80)	0.31 (0.13–0.73)	0.30 (0.12–0.73)	0.51 (0.14–1.78)
High (>80)	0.26 (0.10–0.68)	0.25 (0.09–0.69)	0.41(0.11–1.54)
Age (1 year)		0.99 (0.95–1.04)	0.99 (0.94–1.04)
Functional status			
Good			1.00 (ref)
Less good			2.03 (0.61–6.68)
			
*Group II (n*=*123)*			
Calcidiol (nmol l^−1^)			
Low (<50) Medium	1.00 (ref)	1.00 (ref)	1.00 (ref)
(50–80)	0.73 (0.24-2.24)	1.14 (0.24–5.36)	1.05 (0.21–5.13)
High (>80)	0.57 (0.17–1.91)	0.62 (0.11–3.57)	0.59 (0.10–3.47)
Age (1 year)		0.92 (0.85–1.00)	0.93 (0.86–1.00)
Functional status			
Good			1.00 (ref)
Less good			1.17 (0.88–1.54)
Differentiation grade^a^			
High			1.00 (ref)
Moderate			1.60 (0.35–7.34)
Low			4.91 (0.81–29.8)

a Differentiation grade of tumour tissue; WHO three-grade system.

**Table 3:** The estimated relationship between serum calcidiol and death from prostate cancer among patients receiving hormone therapy (*n*=97)

**Variables**	**Model I RR (95%CI)**	**Model II RR (95%CI)**	**Model III RR (95%CI)**
Calcidiol (nmol l^−1^)			
Low	1.00 (ref)	1.00 (ref)	1.00 (ref)
Medium	0.39 (0.19–0.81)	0.35 (0.17–0.73)	0.18 (0.07–0.46)
High	0.29 (0.12–0.68)	0.20 (0.08–0.50)	0.09 (0.03–0.27)
Group status		0.08 (0.04–0.16)	0.06 (0.02–0.13)
Age (1 year)			1.00 (0.94–1.03)
Functional status			
Good			1.00 (ref)
Less good			1.19 (1.04–1.61)
Differentiation grade^a^			
High			1.00 (ref)
Moderate			0.85 (0.23–3.18)
Low			5.63 (1.42–22.3)

a Differentiation grade of tumour tissue; WHO three-grade system.

**Table 4:** The estimated relationship between serum calcidiol and death from all causes (*n*=160)

**Variables**	**Model I RR (95%CI)**	**Model II RR (95%CI)**	**Model III RR (95%CI)**
Calcidiol (nmol l^−1^)			
Low	1.00 (ref)	1.00 (ref)	1.00 (ref)
Medium	0.41 (0.21–0.79)	0.37 (0.19–0.73)	0.40 (0.20–0.78)
High	0.35 (0.17–0.2)	0.23 (0.11–0.51)	0.24 (0.11–0.53)
Group status		0.08 (0.05–0.14)	0.08 (0.05–0.14)
Age (1 year)			1.00 (0.97–1.04)
Functional status			
Good			1.00 (ref)
Less good			1.32 (1.08–1.61)

## References

[bib1] Ahn J, Peters U, Albanes D, Purdue MP, Abnet CC, Chatterjee N, Horst RL, Hollis BW, Huang W-Y, Shikany JM, Hayes RB (2008) Serum vitamin D concentration and prostate cancer risk: A nested case-control study. J Natl Cancer Inst 100: 796–804 18505967 10.1093/jnci/djn152 PMC3703748

[bib2] Al-Delaimy WK, jansen EHJM, Peeters PHM, Van Der Laan JD, Van Noord PAH, Boshuizen HC, Van Der Schouw YT, Jenab M, Ferarri P, Bueno-de-Mesquita HB (2006) Reliability of biomarkers of iron status, blood lipide, oxidative stress, vitamin D, C-reactive protein and fructosamine in two Dutch cohorts. Biomarkers 11: 370–382 16908443 10.1080/13547500600799748

[bib3] Apperly FL (1941) The relation of solar radiation to cancer mortality in North America. Cancer Res 1: 191–195 10.1158/0008-5472.CAN-15-3169 26773094

[bib4] Berwick M, Armstrong BK, Ben-Porat L, Kricker A, Eberle C, Barnhill R (2005) Sun exposure and mortality from melanoma. J Natl Cancer Inst 97: 195–199 15687362 10.1093/jnci/dji019

[bib5] Bischoff-Ferrari HA, Giovannucci E, Willett WC, Dietrich T, Watson-Hughes B (2006) Estimation of optimal serum concentration of 25-hydroxyvitamin D for multiple health outcomes. Am J Clin 84: 18–28 10.1093/ajcn/84.1.18 16825677

[bib6] Bolland MJ, Grey AB, Ames RW, Horne AM, Mason BH, Wattie DJ, Gamble GD, Bouillon R, Reid IR (2007) Age-, gender- and weight-related effects on levels of 25-hydroxyvitamin D are not mediated by vitamin D binding protein. Clin Endocrinol 67: 259–264 10.1111/j.1365-2265.2007.02873.x 17547688

[bib7] Deeb KK, Trump DL, Johnson CS (2007) Vitamin D signalling pathways in cancer: Potential for anticancer therapeutics. Nat Rev Cancer 7: 684–700, doi: 10.1038/nrc.2196 17721433 10.1038/nrc2196

[bib8] Dunlap N, Schwartz GG, Eads D, Cramer SD, Sherk AB, John V, Koumenis C (2003) 1*α*,25-Dihydroxyvitamin D_3_ (calcitriol) and its analogue, 19-nor-1*α*,25(OH)_2_D_2_, potentiate the effect of ionising radiation on humane prostate cancer cells. Brit J Cancer 89: 746–753 12915889 10.1038/sj.bjc.6601161 PMC2376931

[bib9] Freedman DM, Dosemeci M, McGlynn K (2002) Sunlight and mortality from breast, ovarian, colon, prostate, and non-melanoma skin cancer: a composite death certificate based case-control study. Occup Environ Med 59: 257–262, doi:10.1093/jnci/djm.204 11934953 10.1136/oem.59.4.257 PMC1740270

[bib10] Freedman DM, Looker AC, Chang SC, Graubard BI (2007) Prospective study of serum vitamin D and cancer mortality in the United States. J Natl Cancer Inst 99: 1594–1602 17971526 10.1093/jnci/djm204

[bib11] Garland CF, Garland FC (1980) Do sunlight and vitamin D reduce the likelihood of colon cancer? Int J Epidemiol 9: 227–231 7440046 10.1093/ije/9.3.227

[bib12] Giovannucci E (2005) The epidemiology of vitamin D and cancer incidence and mortality: a review (US). Cancer Causes Control 16: 83–95 15868450 10.1007/s10552-004-1661-4

[bib13] Gorham ED, Garland CF, Garland FC, Grant WB, Mohr SB, Lipkin M, Newmark HL, Giovannucci E, Wei M, Holick MF (2007) Optimal vitamin D status for colorectal prevention, a quantitative meta analysis. Am J Prev Med 32: 210–216 17296473 10.1016/j.amepre.2006.11.004

[bib14] Grant WB (2002) An estimate of premature cancer mortality in the US due to inadequate doses of solar ultra-violet-B radiation. Cancer 94: 1867–1875 11920550 10.1002/cncr.10427

[bib15] Greenspan SL (2008) Approach to the prostate cancer patient with bone disease. J Clin Endocrinol Metab 93: 2–7 10.1210/jc.2007-1402 PMC2190743 18178905

[bib16] Hansen CM, Binderup L, Hamberg KJ, Carlberg C (2001) Vitamin D and cancer: effects of 1,25(OH)_2_D_3_ and its analogs on growth control and tumor genesis. Front Bioski 6: D820–D848 10.2741/hansen 11438443

[bib17] Holick MF (2006) Vitamin D; its role in cancer prevention and treatment. Prog Biophys Mol Biol 92: 49–559 16566961 10.1016/j.pbiomolbio.2006.02.014

[bib18] Holick MF (2007) Vitamin D deficiency. N Engl J Med 19: 357: 266–281 10.1056/NEJMra070553 17634462

[bib19] John EM, Schwartz GG, Dreon DM, Koo J (1999) Vitamin D and breast cancer risk: The NHANES I Epidemiological follow-up study, 1971–1975 to 1992. Cancer Epidemiol Biomark Prev 8: 399–406 10350434

[bib20] Johnsen B, Mikkelborg O, Hannevik M, Nilsen LT, Saxebøl G, Blaasaas KG (2002) The Norwegian UV monitoring program period 1995/96 to 2001. Radiation Protection Authority: Østerås, Norway, pp 23–24

[bib21] Lim HS, Roychoudhuri R, Peto J, Schwartz G, Baade P, Møller H (2006) Cancer survival is dependent on season of diagnosis and sunlight exposure. Int J Cancer 119: 1530–1536 16671100 10.1002/ijc.22052

[bib22] Lissner D, Mason RS, Posen S (1981) Stability of vitamin D metabolites in human blood serum and plasma. Clin Chem 27: 773–774 7226510

[bib23] Luscombe CL, Fryer AA, French ME (2001) Exposure to ultraviolet radiation: association with susceptibility and age at presentation with prostate cancer. Lancet 358: 641–642 11530156 10.1016/S0140-6736(01)05788-9

[bib24] Moan J, Porojnicu AC (2006) The photobiology of vitamin D—a topic of renewed focus. Tidskr Nor Lægefor 126: 1048–1052, (in Norwegian) 16619064

[bib25] Mostofi FK (1980) Histological typing of prostate tumors. International classification of tumors No. 22, World Health Organization: Geneva

[bib26] Ng K, Meyerhardt JA, Wu K, Feskanich D, Hollis BW, Giovannucci EL, Fuchs CS (2008) Circulating 25-Hydroxvitamin D levels and survival in patients with colorectal cancer. J Clin Oncol 36: 2984–2991 10.1200/JCO.2007.15.1027 18565885

[bib27] Nyomba BL, Bouillon R, De MP (1987) Evidence for an interaction of insulin and sex steroids in the regulation of vitamin D metabolism in the rat. J Endocrinol 115: 295–301 2963888 10.1677/joe.0.1150295

[bib28] Oken MM, Creech RH, Tormey DC, Horton J, Davis TE, McFadden ET, Carbone PP (1982) Toxicity and response criteria of the eastern cooperative oncology group. Am J Clin Oncol 5: 649–655 7165009

[bib29] Omdahl JL, Morris HA, May BK (2002) Hydroxylase enzymes of the vitamin D pathway: expression, function, and regulation. Annu Rev Nutr 22: 139–166 12055341 10.1146/annurev.nutr.22.120501.150216

[bib30] Porojnicu AC, Robsahm TE, Ree AH, Moan J (2005) Season of prognosis is a prognostic factor in Hodgkin's lymphoma: a possible role of sun-induced vitamin D. Br J Cancer 93: 571–574 16136030 10.1038/sj.bjc.6602722 PMC2361596

[bib31] Porojnicu AC, Robsahm TE, Dahlback A, Berg JP, Christiani D, Bruland OS, Moan J (2007) Seasonal and geographical variations in lung cancer prognosis in Norway. Does vitamin D from the sun play any role? Lung Cancer 55: 263–270, doi: 10.1016/j.lungcan.2006.11.013 17207891 10.1016/j.lungcan.2006.11.013

[bib32] Robsahm TE, Tretli S, Dahlback A, Moan J (2004) Vitamin D from sunlight may improve the prognosis of breast-, colon- and prostate cancer (Norway). Cancer Causes Control 2: 149–158 10.1023/B:CACO.0000019494.34403.09 15017127

[bib33] Sarem K, Pedersen JI (1988) Sex differences in the hydroxylation of cholecalciferol and of 5 beta-cholestane-3 alpha, 7 alpha, 12 alpha-triol in rat liver. Biochem J 247: 73–78 10.1042/bj2470073 PMC1148371 2825658

[bib34] Schwartz GG, Hulka BS (1990) Is vitamin D deficiency a risk factor for prostate cancer? Hypothesis). Anticancer Res 10: 1307–1311 2241107

[bib35] Schwartz GG, Whitlatch LW, Chen TC, Lokeshwar BL, Holick MF (1998) Human prostate cells synthesize 1,25- hydroxyvitamin D_3_ from 25-hydroxyvitamin D_3_. Cancer Epidemiol Biomark Prev 7: 391–395 9610788

[bib36] Schwartz GG (2005) Vitamin D and the Epidemiology of Prostate Cancer. Seminars in Dialysis 18: 276–289 16076349 10.1111/j.1525-139X.2005.18403.x

[bib37] SPSS for Windows, version 15.0, Chicago, Illnois, Spss Inc, 2007

[bib38] Tuohimaa P, Tenkanen L, Ahonen M, Lumme S, Jellum E, Hallmans G, Stattin P, Harvei S, Hakulinen T, Luostarinen T, Dillner J, Lethinen M, Hakama M (2004) Both high and low levels of blood vitamin D are associated with a higher prostate cancer risk: a longitudinal, nested case-control study in the nordic countries. Int J Cancer 108: 104–108 14618623 10.1002/ijc.11375

[bib39] Vieth R (2006) What is the optimal vitamin D status for health. Prog Biophys Mol Biol 92: 26–32, doi: 10.1016/j.pbiomolbio.2006.02.003 16766239 10.1016/j.pbiomolbio.2006.02.003

[bib40] Wolpowitz D, Gilchrest BA (2006) The vitamin D questions: how much do you need and how should you get it? J Am Acad Dermatol 54: 301–317 16443061 10.1016/j.jaad.2005.11.1057

[bib41] Woo TCS, Choo R, Jamieson M, Chander S, Vieth R (2005) Pilot study: potential role of vitamin D (cholecalciferol) in patients with PSA relapse after definitive therapy. Nutr Cancer 51: 32–36 15749627 10.1207/s15327914nc5101_5

[bib42] Zhou W, Heist RS, Liu G, Asomaning K, Neuberg DS, Hollis BW, Wain JC, Lynch TJ, Giovannucci E, Su L, Christiani DC (2007) Circulating 25-hydroxyvitamin D levels predict survival in early-stage non-small lung cancer patients. J Clin Oncol 25: 479–485 17290055 10.1200/JCO.2006.07.5358

